# Supplementary material for: Gold Nanoparticles Induced Size Dependent Cytotoxicity on Human Alveolar Adenocarcinoma Cells by Inhibiting the Ubiquitin Proteasome System
Source: Pharmaceutics. 2023 Jan 28;15(2):432. doi: 10.3390/pharmaceutics15020432 (PMC9961554; doi:10.3390/pharmaceutics15020432)
Supplement: Supplementary file 1 [file pharmaceutics-15-00432-s001.zip › pharmaceutics-2128033-supplementary.pdf]

Gold nanoparticles induced size dependent cytotoxicity on human alveolar adenocarcinoma cells by inhibiting the ubiquitin proteasome system

Bashiru Ibrahim[1,2], Taiwo Hassan Akere[1,2], Swaroop Chakraborty[2], Eugenia Valsami-Jones[2*] and Hanene Ali-Boucetta[2*]

[1]Nanomedicine, Drug Delivery & Nanotoxicology (NDDN) Lab, School of Pharmacy, College of Medical & Dental Sciences, University of Birmingham, Birmingham, B15 2TT United Kingdom

[2]FENAC Lab, School of Geography, Earth and Environmental Sciences, College of Life and Environmental Sciences, University of Birmingham, Birmingham B15 2TT, United Kingdom

Corresponding author e-mail: : h.aliboucetta@bham.ac.uk ; e.valsamijones@bham.ac.uk

Supplementary Information

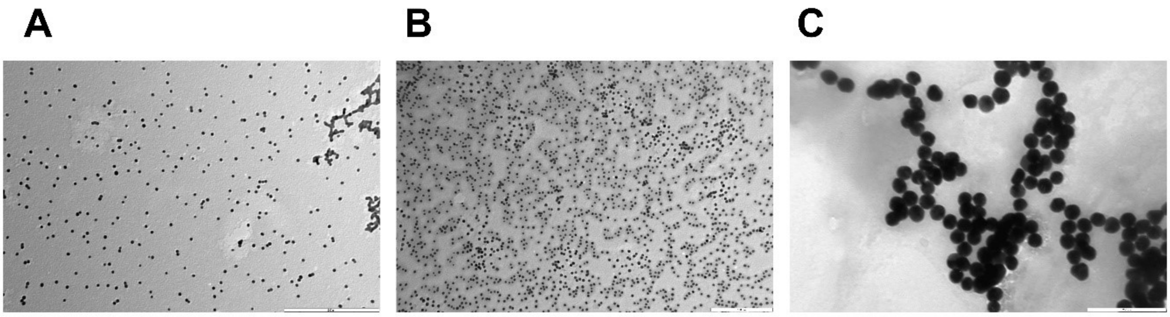

Figure S1. Characterisation of different sizes AuNPs dispersed in Ultrapure water by Transmission of electron microscopy (TEM): (A) 5 nm AuNPs (B) 10 nm AuNPs (C) 80 nm AuNPS

Surface area (SA)

The surface area of different size AuNPs corresponding to the concentrations used in the cell viability assay was calculated according to Teeguarden *et al.*, (2007) using the below equation. Where AuNPs are assumed to be spherical or nanosphere, r is the radius of AuNPs suspended in medium determined by DLS and V is the volume of a sphere used [68].

$$V=4/3(\lambda * r^3)$$

For instance, the surface area of 5 nm AuNPs (17.57 nm diameter measured with DLS) at a starting concentration was as follows.

Mass concentration (gm)	concentration	0.005
Diameter (nm)	r^{*2}	308.7049
Radius (r)	Diameter/2	154.35245
Density of Au (g/cm^3)	19.32	19.32

Volume (V)	mass/density	0.000258799
Volume per particle (v)	4/3(л*r³) → $4/3(л*r^3)$	15011136.78
N	V/v	1.72405E-11
SA per particles (M^2)	$4*л*r^2$	299237.966
Total SA M^2/g	N*sa (m^2)	5.15901E-06

Table S1: Surface area (m^2/g) of different 5, 10 and 80 nm AuNPs at different mass concentrations used in the cell viability assays.

	Mass concentration (µg/mL)							
AuNPs	5	10	15	20	25	30	35	40
5 nm	5.13E-06	1.03E-05	1.55E-05	2.06E-05	2.58E-05	3.1E-05	3.61E-05	4.13E-05
10 nm	1.36E-06	2.73E-06	4.09E-06	5.46E-06	6.82E-06	8.18E-06	9.55E-06	1.09E-05
80 nm	1.27E-07	2.54E-07	3.81E-07	5.08E-07	6.35E-07	7.62E-07	8.89E-07	1.02E-06

Particle number concentration

The particles number concentration of AuNPs was calculated according to the formula described by Huk *et al.*, [69]. The mass (Au) concentration(mg/mL) and the particle concentration (NPs/ml) of different size AuNPs were provided in the certificate of analysis of NPs by manufacturer (https://tools.nanocomposix.com:48/cdn/coa/Gold/Spheres/NanoXact/AU5-NX-CIT-JRC0024.pdf?121%20956). Using the highest concentration of AuNPs used for our study (40 µg/mL), we calculated the particle number concentration using the formula below:

PNC= NPs mass concentration * NPs/mL/Mass Au

Table S2: The table show the number of particles per volume (NPs/ML) for the highest concentration used in the cell viability assay and the number of viable cells remained after exposed to different size AuNPs for 24 h.

AuNPs	40 µg/mL	
	NPs/mL	% Viability
5 nm	3.17E+13	9.240198
10 nm	2.50E+12	12.9125
80 nm	8.46E+09	29.08156

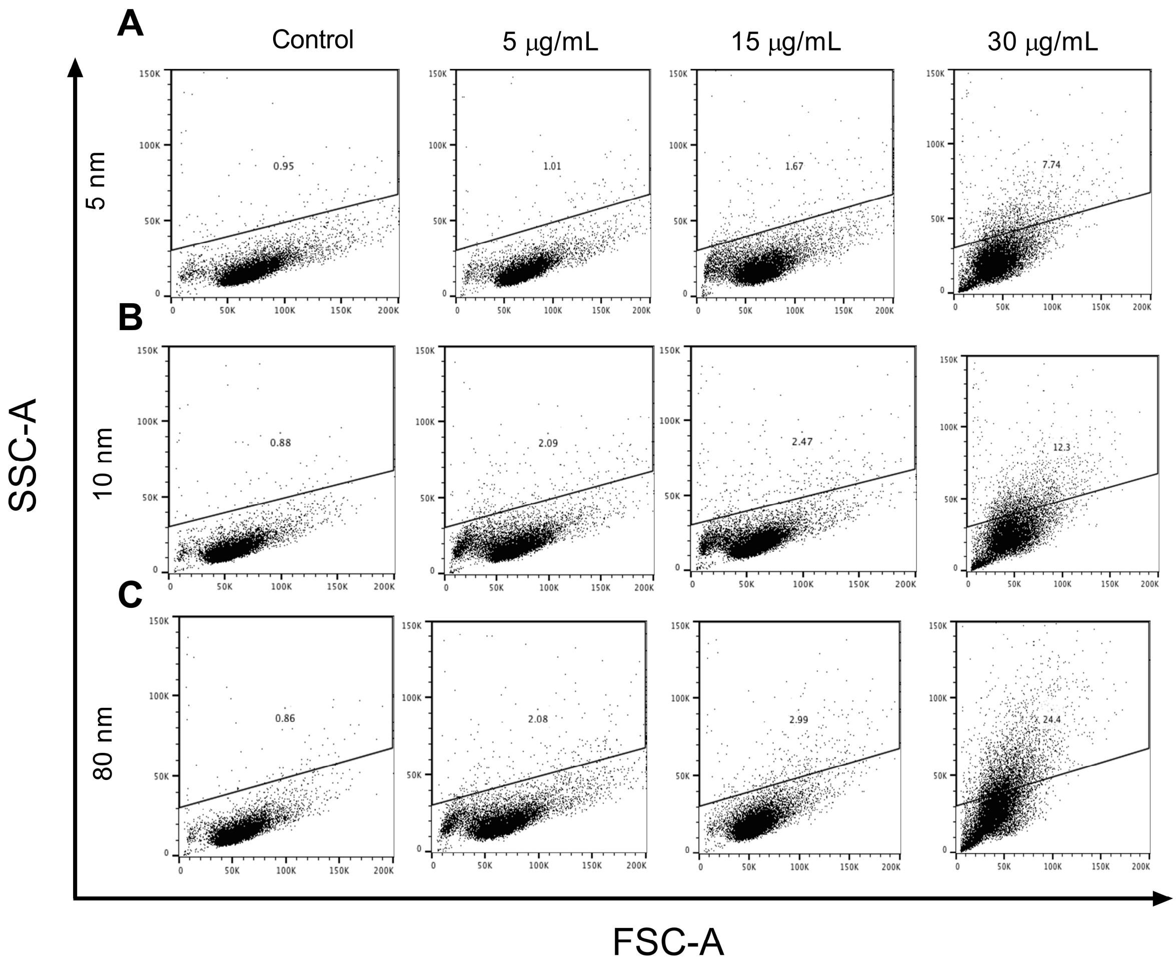

Figure S2. Dot plots of side scatted intensity (SSC) versus forward scattered intensity (FSC) of A549 cells treated with different concentration of AuNPs for 24 h. (A) 5 nm (B) 10 nm (C) 80 nm

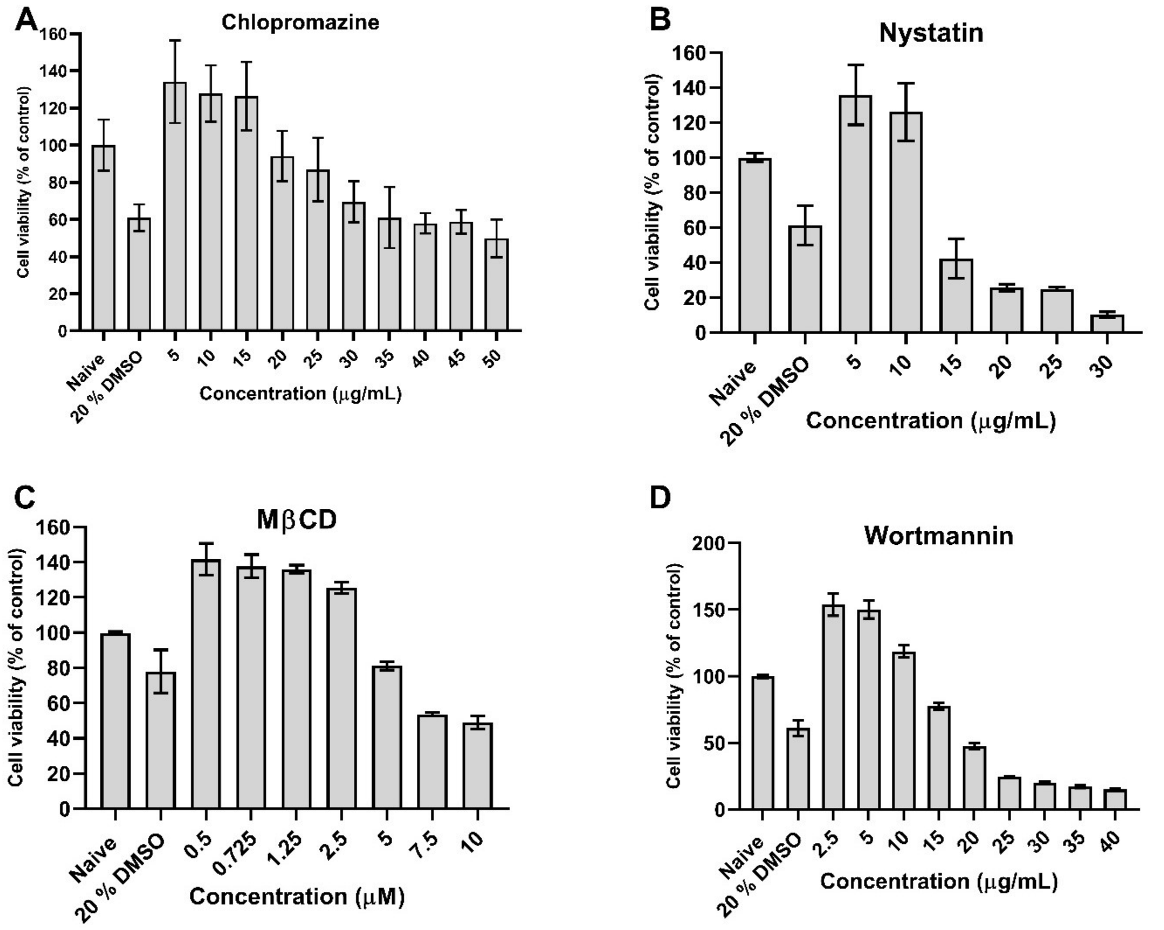

Figure S3: Percentage cell viability of A549 cells determined using MTT assay for pre-treated with pharmacological inhibitors (**A**) Chlorpromazine hydrochloride, inhibitor of Clathrin mediated endocytosis (**B**) Nystatin, inhibitor of Caveolae mediated endocytosis (**C**) MβCD, inhibitor of Caveolae mediated endocytosis (**D**) Wortmannin, inhibitor of macropinocytosis for different concentration after 1 h exposure. 20% DMSO was used as a positive control.

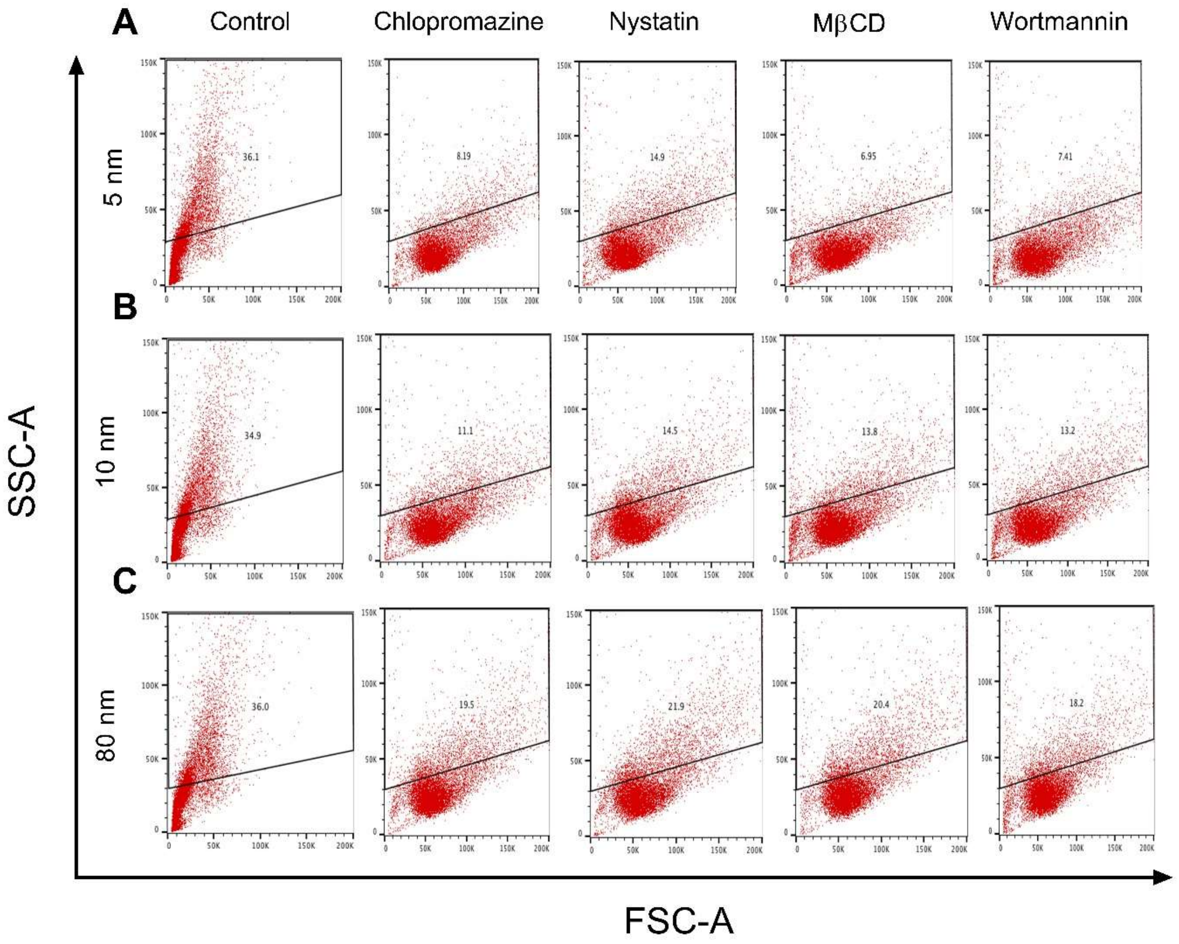

Figure S4: Dot plots effects of pharmacological inhibitors on the internalisation of different size AuNPs in A549 cells. Cells were separately pre-incubated with Chlorpromazine, Nystatin, methyl-β-cyclodextrin (MβCD) and Wortmannin for 1 h, followed by adding AuNPs incubation for 3 h and analysed flow cytometry. (A) 5 nm (B) 10 nm (C) 80 nm

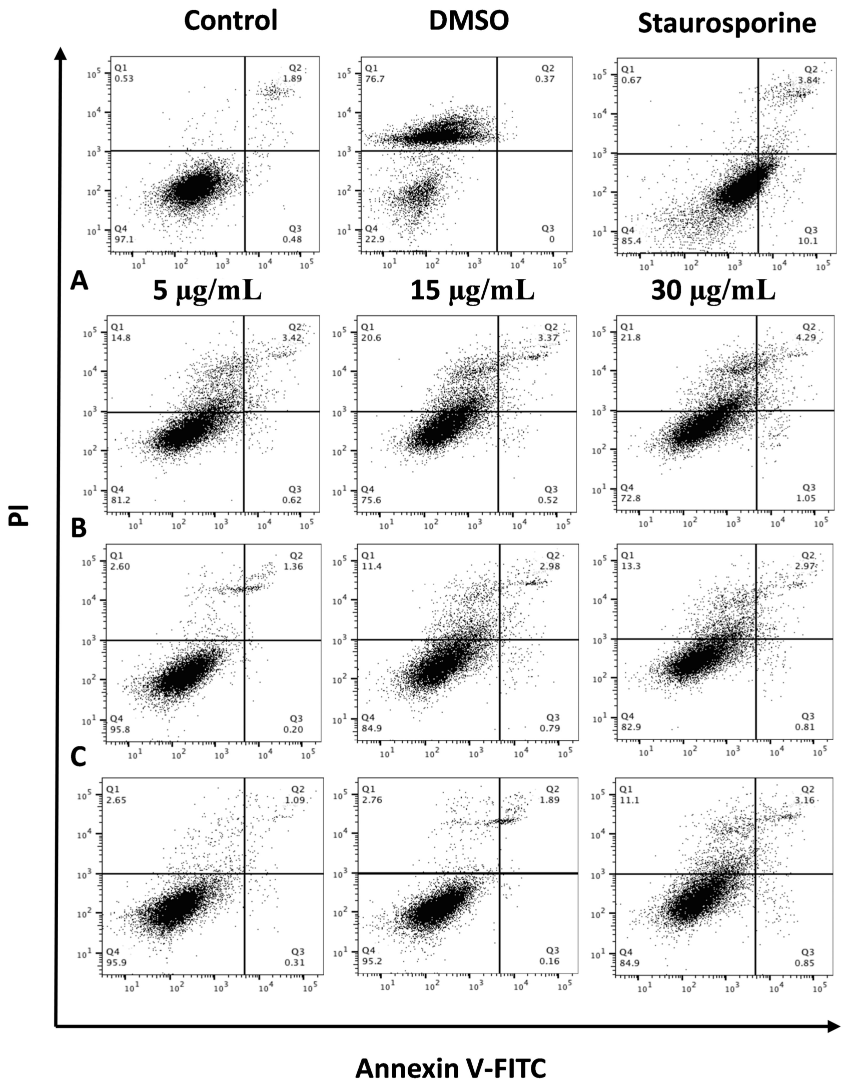

Figure S5. Apoptosis and necrosis analysis of A549 cells following the exposure to AuNPs of different sizes flow cytometry at of 5-30 µg/mL for 24 h**.** (A) 5 nm (B) 10 nm (C) 80 nm. 1 µM Staurosporine and 10 % DMSO was used as positive control for apoptosis and necrosis for 6 h.
